# Long-Term Psychiatric Outcomes in Adult Patients With Migraine on Prophylactic Adjunctive Amitriptyline vs. Duloxetine: A Retrospective Cohort TriNetX Study

**DOI:** 10.7759/cureus.107916

**Published:** 2026-04-28

**Authors:** Manasa Mula, Zachary Li, Taylor M Gong, Eduardo D Espiridion

**Affiliations:** 1 Medicine, Drexel University College of Medicine, Wyomissing, USA; 2 Psychiatry, Drexel University College of Medicine, Wyomissing, USA

**Keywords:** anxiety, depression, duloxetine vs amytriptyline, migraine headaches, prophylactic migraine therapy, trinetx

## Abstract

Migraine, a primary headache disorder, is a prevalent and debilitating chronic neurologic disorder. Migraine headaches are bidirectionally related to psychiatric conditions, such as anxiety and depression. Given these associations, further investigation into how medications used for migraine prophylaxis are associated with long-term psychiatric outcomes is warranted. Our study aims to examine, among patients with migraine, whether the use of adjunctive antidepressants (duloxetine or amitriptyline) in combination with topiramate is associated with differences in the incidence of long-term psychiatric outcomes, such as anxiety and depression.

A retrospective cohort study with a five-year follow-up period was performed using TriNetX, a global federated health research network, and included patients aged ≥18 years with migraine who were started on either topiramate + duloxetine or topiramate + amitriptyline within one month on or after the first instance of documented migraine diagnosis in their electronic health record. After propensity score matching, both groups comprised 1241 patients. We found that patients on prophylactic topiramate + duloxetine had significantly greater five-year risks of anxiety disorder (unspecified), bipolar disorder, depressive episode, major depressive disorder, panic disorder, and post-traumatic stress disorder compared to patients on prophylactic topiramate + amitriptyline (p<0.05). In contrast, the risk of generalized anxiety disorder did not significantly differ between the two groups.

There is limited data comparing duloxetine and amitriptyline when used for migraine prophylaxis in relation to subsequent psychiatric outcomes. Our study helps address this gap using real-world observational evidence. Our study may help inform future randomized clinical trials directly comparing these antidepressants for migraine prophylaxis, as well as provide a basis for future research on treatment selection, considering patients’ severity of neurological symptoms, psychiatric risk factors, tolerability of adverse effects, and potential treatment benefits.

## Introduction

Migraine, a primary headache disorder, is a debilitating chronic condition affecting more than one billion people globally as the second most prevalent neurologic disorder [1-3]. The disease burden of migraine has been significantly rising since 1990, and according to the Global Burden of Disease Study 2021, migraine is among the top 10 conditions of the nervous system with the highest disability-adjusted life-years [2,4]. 

In addition to its disabling neurologic symptoms, such as unilateral, pulsating headaches, moderate-to-severe pain, nausea, vomiting, photophobia, and phonophobia, migraine headaches are bidirectionally related to mood disorders such as anxiety and depression [1,5,6]. Literature suggests that patients with depression have an earlier onset and increased severity of migraine headaches, and patients with migraine have a 2.5 times greater risk of developing depression, suggesting shared biological mechanisms between these two conditions [7]. Additionally, patients with migraine have 3.76-fold higher odds of panic disorder and 3.07-fold higher odds of post-traumatic stress disorder (PTSD) [8,9]. The diagnoses of migraine and bipolar disorder are also associated with studies showing that around 20% of migraineurs have comorbid bipolar disorder [10-12]. Given these associations, further investigation into how medications used for migraine prophylaxis are associated with long-term psychiatric outcomes is warranted.

Currently, several classes of drugs are utilized for migraine prophylaxis, including antihypertensives (e.g., propranolol, metoprolol), antiepileptics (e.g., topiramate, sodium valproate), and antidepressants (e.g., amitriptyline, venlafaxine, duloxetine) [13]. Recent literature has shown that while selective norepinephrine reuptake inhibitors (SNRIs) have similar efficacy compared to tricyclic antidepressants (TCAs) in reducing the number of migraine attacks per month, SNRIs have fewer adverse events [14]. 

However, there is limited data comparing these two antidepressant classes (specifically, duloxetine and amitriptyline) when used for migraine prophylaxis in relation to subsequent psychiatric outcomes such as anxiety and depression. A better understanding of these associations may inform future research studies and ultimately clinical decision-making. Our study aims to examine, among patients with migraine, whether the use of adjunctive antidepressants (duloxetine or amitriptyline) in combination with topiramate is associated with differences in the incidence of long-term psychiatric outcomes, such as anxiety and depression.

## Materials and methods

Data source

A retrospective study was performed using the TriNetX, LLC platform, a global federated health research network. The data used in this study was collected on September 22, 2025 from the TriNetX Research Network, which provided access to electronic medical records (diagnoses, procedures, medications, laboratory values, genomic information) from approximately 157 million patients from 108 healthcare organizations (HCOs). Institutional Review Board approval was not required [15], and this study was exempt from informed consent. The data reviewed was a secondary analysis of existing data, did not involve intervention or interaction with human subjects, and was de-identified per the de-identification standard defined in Section §164.514(a) of the HIPAA Privacy Rule. The process by which the data was de-identified is attested to through a formal determination by a qualified expert as defined in Section §164.514(b)(1) of the HIPAA Privacy Rule. This formal determination by a qualified expert was refreshed on December 2020. Within the TriNetX platform, International Classification of Diseases, Tenth Revision, Clinical Modification (ICD-10-CM) codes were utilized to identify diagnoses, covariates, and outcomes. 

Study population and design 

**Figure 1:**
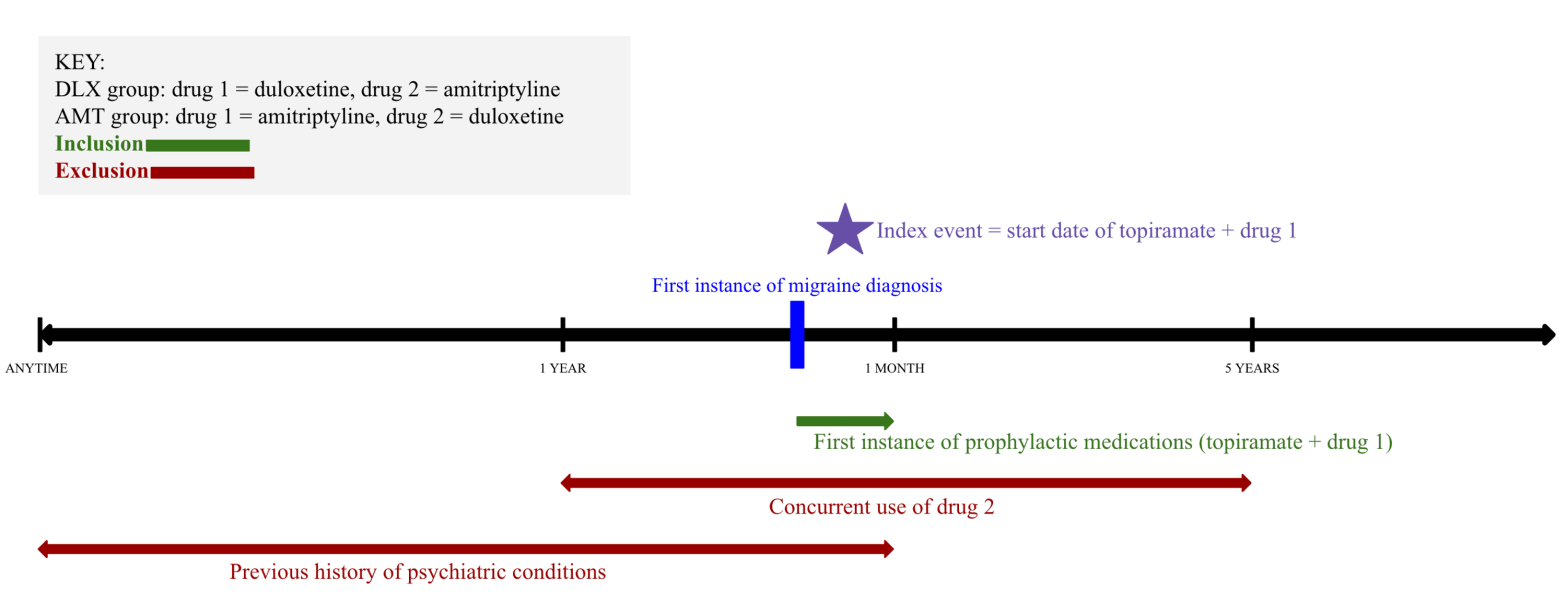
Study Design

This retrospective cohort study with a five-year follow-up period included patients aged ≥18 years with migraine (ICD10 G43) diagnosed between September 1, 2010 and September 1, 2020 who were started on either topiramate and duloxetine (DLX group) or topiramate and amitriptyline (AMT group) within one month on or after the first instance of documented migraine diagnosis in their electronic health record (Figure 1). This medication framework on TriNetX was used as a proxy to study migraine prophylaxis involving adjunctive use of duloxetine and amitriptyline alongside topiramate, an established first-line and efficacious migraine prophylactic agent [16]. Patients in the DLX and AMT groups were excluded if they had documented amitriptyline or duloxetine use, respectively, within 1 year before or up to five years after the first migraine diagnosis (Figure 1). This approach excludes patients who received the comparator medication at any point during the follow-up period, modeling a non-switching treatment population, rather than a traditional intent-to-treat analysis. Further exclusion criteria included diagnosis with the following psychiatric conditions anytime before or up to one month after the first migraine diagnosis: anxiety disorder, unspecified (ICD10 F41.9); bipolar disorder (ICD10 F31); depressive episode (ICD10 F32); generalized anxiety disorder (ICD10 F41.1); major depressive disorder, recurrent (ICD10 F33); panic disorder (episodic paroxysmal anxiety) (ICD10 F41.0); and PTSD (ICD10 F43.1) (Figure 1). The index event was defined as the date when prophylactic treatment with both medications (either topiramate + duloxetine or topiramate + amitriptyline) was initiated within one month on or after the first migraine diagnosis (Figure 1). Topiramate and the respective prophylactic treatment (duloxetine or amitriptyline) could be initiated at any time within the one-month window, with day 0 defined as the date of the second prescription, completing the combination regimen. 

Statistical analysis

Propensity score matching (PSM) was performed to control for baseline differences between the DLX and AMT groups. Based on baseline covariates, a propensity score, ranging from 0 to 1, was generated for each patient using a logistic regression model. Based on the calculated propensity scores, a 1:1 greedy, nearest-neighbor matching with a caliper of 0.1 pooled standard deviations was performed to yield an equal number of patients in both groups. Age at index, sex, race, and ethnicity were included in PSM. Furthermore, PSM was performed on the following FDA-approved or off-label indications for duloxetine and amitriptyline [17,18]: type 2 diabetes mellitus with diabetic neuropathy, unspecified (ICD10 E11.40), type 2 diabetes mellitus with diabetic polyneuropathy (ICD10 E11.42), chronic pain syndrome (ICD10 G89.4), fibromyalgia (ICD10 M79.7), interstitial cystitis (chronic) (ICD10 N30.1), insomnia, unspecified (ICD10 G47.00), irritable bowel syndrome (ICD10 K58), postherpetic trigeminal neuralgia (ICD10 B02.22), myalgia (ICD10 M79.1), and low back pain (ICD10 M54.5). Standardized mean differences (SMDs) less than 0.1 indicated negligible differences between groups for the respective covariate [19]. 

The primary outcomes of interest were anxiety disorder, unspecified (ICD10 F41.9), bipolar disorder (ICD10 F31), depressive episode (ICD10 F32), generalized anxiety disorder (ICD10 F41.1), major depressive disorder, recurrent (ICD10 F33), panic disorder (episodic paroxysmal anxiety) (ICD10 F41.0), and post-traumatic stress disorder (PTSD) (ICD10 F43.1). Five-year psychiatric outcomes were compared between the DLX and AMT groups using risk differences (RDs), risk ratios (RRs), odds ratios (ORs), and their corresponding p-values or 95% confidence intervals (CIs). All statistical analyses were obtained from the TriNetX analytics tool. A p-value of less than 0.05 was considered statistically significant. Given the observational design, the results are presented as associations and hypothesis-generating. 

## Results

After applying the inclusion and exclusion criteria, the DLX group contained 1318 patients, and the AMT group contained 2653 patients. Due to the dynamic nature of the TriNetX platform, with data being frequently updated by HCOs, PSM was performed on 1315 patients in the DLX group and 2624 patients in the AMT group at the time the analysis was run. After PSM, the DLX and AMT groups each comprised 1241 patients. The covariates listed in Table 1 were well-balanced between the two groups as standardized mean differences were less than 0.1. In the DLX group, the mean age (SD) was 45.7 (12.5). Of the patients in the DLX group, 1092 (88.0%) were female, 105 (8.5%) were Black or African American, 53 (4.3%) were Hispanic or Latino, and 897 (72.3%) were White. In the AMT group, the mean age (SD) was 45.8 (12.7). Of the patients in the AMT group, 1109 (89.4%) were female, 101 (8.1%) were Black or African American, 42 (3.4%) were Hispanic or Latino, and 920 (74.1%) were White. 

**Table 1:** Baseline Patient Characteristics Before and After Propensity Score Matching PSM: propensity score matching; DLX topiramate + duloxetine group; AMT topiramate + amitriptyline group; Std diff.: standardized (mean) difference, SD: standard deviation; T2DM: type 2 diabetes mellitus.

Characteristic	Before PSM, No. (%)			After PSM, No. (%)		
	DLX (n = 1315)	AMT (n = 2624)	Std diff.	DLX (n = 1241)	AMT (n = 1241)	Std diff.
Age at index, mean ± SD	46.5 ± 12.8	40.1 ± 13.1	0.492	45.7 ± 12.5	45.8 ± 12.7	0.008
Female	1163 (88.4)	2165 (82.5)	0.169	1092 (88.0)	1109 (89.4)	0.043
Male	116 (8.8)	396 (15.1)	0.194	115 (9.3)	104 (8.4)	0.031
Asian	10 (0.8)	123 (4.7)	0.243	10 (0.8)	10 (0.8)	<0.001
Black or African American	106 (8.1)	342 (13.0)	0.162	105 (8.5)	101 (8.1)	0.012
Hispanic or Latino	53 (4.0)	181 (6.9)	0.126	53 (4.3)	42 (3.4)	0.046
White	960 (73.0)	1698 (64.7)	0.180	897 (72.3)	920 (74.1)	0.042
T2DM with diabetic neuropathy, unspecified	11 (0.8)	10 (0.4)	0.059	10 (0.8)	10 (0.8)	<0.001
T2DM with diabetic polyneuropathy	10 (0.8)	10 (0.4)	0.050	10 (0.8)	10 (0.8)	<0.001
Chronic pain syndrome	10 (0.8)	11 (0.4)	0.045	10 (0.8)	10 (0.8)	<0.001
Fibromyalgia	86 (6.5)	64 (2.4)	0.199	54 (4.4)	44 (3.5)	0.041
Interstitial cystitis (chronic)	10 (0.8)	10 (0.4)	0.050	10 (0.8)	10 (0.8)	<0.001
Insomnia, unspecified	20 (1.5)	54 (2.1)	0.041	18 (1.5)	15 (1.2)	0.021
Irritable bowel syndrome	26 (2.0)	40 (1.5)	0.035	22 (1.8)	21 (1.7)	0.006
Postherpetic trigeminal neuralgia	0 (0)	0 (0)	--	0 (0)	0 (0)	--
Myalgia	50 (3.8)	78 (3.0)	0.046	41 (3.3)	38 (3.1)	0.014
Low back pain	68 (5.2)	150 (5.7)	0.024	63 (5.1)	46 (3.7)	0.067

The five-year risk of psychiatric outcomes (Table 2) was significantly higher in the DLX group compared to the AMT group for several diagnoses. The most common outcome was depressive episode, which occurred in 251 (20.23%) patients in the DLX group and 181 (14.59%) patients in the AMT group, with an RD of 5.64% (p<.001), RR of 1.387 (95% CI: 1.165, 1.651), and OR of 1.485 (95% CI: 1.204, 1.831). The second most common outcome was anxiety disorder, unspecified, which occurred in 228 (18.37%) patients in the DLX group and 183 (14.75%) patients in the AMT group, with an RD of 3.63% (p=0.02), RR of 1.246 (95% CI: 1.043, 1.488), and OR of 1.301 (95% CI: 1.052, 1.610). Other psychiatric outcomes with elevated risk in the DLX group compared to the AMT group include bipolar disorder (RD 1.45%, p=0.005), major depressive disorder, recurrent (RD 2.34%, p=0.003), panic disorder (RD 0.97%, p=0.04), and post-traumatic stress disorder (RD 1.37%, p=0.02) (Table 2). The only psychiatric outcome that did not significantly differ between the DLX and AMT groups was generalized anxiety disorder, which occurred in 60 (4.84%) patients in the DLX group and 53 (4.27%) patients in the AMT group, with an RD of 0.56% (p=0.50), RR of 1.132 (95% CI: 0.789, 1.624), and OR of 1.139 (95% CI: 0.780, 1.662) (Table 2).

**Table 2:** Five-Year Risk of Psychiatric Outcomes After PSM PSM: propensity score matching; DLX topiramate + duloxetine group; AMT topiramate + amitriptyline group. p-values were calculated using a Z-test.

Outcome	DLX, No. (%) (n = 1241)	AMT, No. (%) (n = 1241)	Risk Difference (%)	Z-value	P-value	Risk Ratio (95% CI)	Odds Ratio (95% CI)
Anxiety disorder, unspecified	228 (18.37)	183 (14.75)	3.63	2.430	0.02	1.246 (1.043, 1.488)	1.301 (1.052, 1.610)
Bipolar disorder	30 (2.42)	12 (0.97)	1.45	2.801	0.005	2.500 (1.286, 4.860)	2.537 (1.293, 4.979)
Depressive episode	251 (20.23)	181 (14.59)	5.64	3.706	<0.001	1.387 (1.165, 1.651)	1.485 (1.204, 1.831)
Generalized anxiety disorder	60 (4.84)	53 (4.27)	0.56	0.674	0.50	1.132 (0.789, 1.624)	1.139 (0.780, 1.662)
Major depressive disorder, recurrent	64 (5.16)	35 (2.82)	2.34	2.975	0.003	1.829 (1.220, 2.740)	1.874 (1.231, 2.851)
Panic disorder (episodic paroxysmal anxiety)	24 (1.93)	12 (0.97)	0.97	2.015	0.04	2.000 (1.005, 3.981)	2.020 (1.005, 4.057)
Post-traumatic stress disorder (PTSD)	35 (2.82)	18 (1.45)	1.37	2.360	0.02	1.944 (1.107, 3.414)	1.972 (1.111, 3.501)

## Discussion

In our retrospective cohort study of patients with migraine, our results suggest that adjunctive amitriptyline, when combined with topiramate, is associated with lower long-term psychiatric morbidity for certain outcomes compared with adjunctive duloxetine. We found that patients on prophylactic topiramate + duloxetine had significantly greater five-year risks of anxiety disorder (unspecified), bipolar disorder, depressive episode, major depressive disorder, panic disorder, and PTSD compared to patients on prophylactic topiramate + amitriptyline. Although several psychiatric outcomes were statistically different between the two groups, the risk differences were small. In a large dataset, since even minor differences can achieve statistical significance, these findings should be interpreted cautiously, as they may not reflect meaningful clinical differences. Nonetheless, there is limited research directly examining the role of adjunctive duloxetine versus amitriptyline in long-term psychiatric outcomes among patients with migraine. Our study contributes to this gap using real-world observational data. 

One possible interpretation of our findings is that patients receiving amitriptyline may experience differences in migraine symptom management and physical pain compared with those receiving duloxetine, which may be associated with variations in long-term psychiatric outcomes. However, this explanation is not well supported by the current literature; Shahid et al. [14] demonstrated that venlafaxine, another SNRI, was associated with shorter migraine attack duration, fewer side effects, and similar migraine attacks per month compared to TCAs, such as amitriptyline and nortriptyline. In other meta-analyses by Banzi et al. [20] and Xu et al. [21], there were no significant differences in migraine frequency, intensity, duration, or prevention between amitriptyline and SNRIs. Although duloxetine and amitriptyline were not compared head-to-head in these three papers, the findings suggest that broadly, TCAs may not be superior to SNRIs in the management of migraine symptoms. Overall, further research directly comparing duloxetine and amitriptyline is needed to better understand our findings, particularly the explanation that amitriptyline may be associated with better psychiatric outcomes due to more effective migraine management.

The observed differences in psychiatric outcomes in our study could be explained by the distinct analgesic and sedative profiles of duloxetine and amitriptyline. In fibromyalgia patients, duloxetine was associated with better treatment of pain and depression, while amitriptyline had improved sleep, fatigue, and quality of life when compared to placebo [22]. These findings help contextualize our results, suggesting that adjunctive amitriptyline use in patients with migraine may be associated with the observed differences in psychiatric outcomes due to its potential influence on sleep and quality of life. In contrast, a study examining chronic diabetic peripheral neuropathic pain discusses how, despite its analgesic efficacy, duloxetine is associated with CNS-activating effects and sleep fragmentation [23]. This finding may explain why patients with migraine receiving adjunctive duloxetine generally had a higher observed incidence of psychiatric outcomes, potentially mediated by disrupted sleep cycles. Collectively, evidence from these papers on other chronic pain conditions suggests that the favorable psychiatric outcomes observed with adjunctive amitriptyline compared to duloxetine in patients with migraine may be explained by the important role of sleep quality (rather than analgesia alone) on long-term pain management and subsequent mental health outcomes.

Our study presents several limitations. Firstly, this study did not account for the dosage, duration, or adherence of migraine prophylactic medications, which can greatly impact the role that these treatments play in preventing future psychiatric outcomes. We also did not control for other treatments, such as different migraine prophylaxis classes or psychotherapies, that the patients were concurrently started on during the follow-up period, which can influence their migraine management and/or development of psychiatric conditions. Along these lines, we did not account for acute migraine abortive medications (e.g., NSAIDs, triptans), which could influence the development of medication-overuse headaches, migraine severity, and psychiatric outcomes. We examined psychiatric outcomes broadly in patients with migraine; however, differences may emerge after stratifying by migraine subtype, such as episodic migraine, chronic migraine, or migraine with aura. Furthermore, we did not adjust for baseline healthcare utilization (e.g., the number of clinical encounters prior to the index date), which serves as a proxy for migraine severity, and patients with higher surveillance may have been more likely to receive psychiatric diagnoses, introducing potential detection bias. Body mass index was not included in PSM and represents an important unmeasured confounder, as duloxetine and amitriptyline have differing effects on weight, and the interplay between obesity, migraine severity, and psychiatric conditions may have influenced our study findings.

Despite PSM, confounding factors such as family history of migraine, undiagnosed childhood mental health issues, and present psychosocial stressors could not be captured and balanced. Furthermore, we utilized ICD-10-CM codes to build the study on TriNetX, which may have inadvertently led to misclassifications or missed diagnoses in our study due to coding errors and/or variations in different clinical practices. Another key limitation is creating a highly selected subgroup of patients through post-index exclusion of those who received the comparator medication, which may introduce selection bias against patients who switched treatments due to treatment failure or adverse effects, limiting the generalizability of our findings. We performed multiple statistical comparisons without applying a Bonferroni correction, which could increase the risk of false-positive errors and warrants cautious interpretation of psychiatric outcomes with p-values near 0.05 (e.g., panic disorder, post-traumatic stress disorder, and anxiety disorder, unspecified). Furthermore, obtaining continuous follow-up data over a five-year period may not be feasible in a health research network, as patients can transfer care between healthcare organizations, change insurance providers, or be lost to follow-up. Future research should address this limitation by employing time-to-event analyses, such as Kaplan-Meier curves or Cox proportional hazards models, which appropriately account for variable follow-up durations and censoring. Given these limitations and the retrospective, observational design of this study, causal relationships between the prophylactic medication combinations and long-term psychiatric outcomes cannot be established; rather, we report exploratory associations.

Our study has several notable strengths. First, the use of TriNetX enabled statistical analysis with a large, diverse sample consisting of real-world data from multiple healthcare organizations, enhancing the generalizability of our results. Furthermore, it allowed for direct focus on duloxetine and amitriptyline in patients with migraine, as opposed to previous literature, which grouped the drugs by class. The robust PSM algorithm ensured well-balanced cohorts for comparison. Additionally, the retrospective nature allowed us to investigate psychiatric outcomes based on how adjunctive duloxetine and amitriptyline are prescribed in routine clinical practice, without requiring randomization. Finally, the five-year follow-up period demonstrates the long-term outcomes of migraine prophylactic combinations.

## Conclusions

In conclusion, our observational findings suggest an association between adjunctive amitriptyline use and lower long-term psychiatric outcomes compared to adjunctive duloxetine in patients with migraine. These results are hypothesis-generating and may help inform future randomized clinical trials directly comparing these antidepressants for migraine prophylaxis, as well as provide a basis for future research on treatment selection, considering patients’ severity of neurological symptoms, psychiatric risk factors, tolerability of adverse effects, and potential treatment benefits.
